# Too hot to reason? Experimental heatwaves affect cognitive traits in male guppies

**DOI:** 10.1093/beheco/araf061

**Published:** 2025-05-30

**Authors:** Merel C Breedveld, Luna Dudine, Samuele Padovan, Marta Giacomazzo, Ranieri Verin, Clelia Gasparini

**Affiliations:** Department of Biology, University of Padova, Via U. Bassi 58/B, Padova 35131, Italy; Department of Biology, University of Padova, Via U. Bassi 58/B, Padova 35131, Italy; Department of Developmental Psychology and Socialisation, University of Padova, Via Venezia 8, Padova 35131, Italy; Department of Biology, University of Padova, Via U. Bassi 58/B, Padova 35131, Italy; Department of Comparative Biomedicine and Food Science, University of Padova, Viale dell’Università 16, Agripolis Legnaro (Padova) 35020, Italy; Department of Comparative Biomedicine and Food Science, University of Padova, Viale dell’Università 16, Agripolis Legnaro (Padova) 35020, Italy; Department of Biology, University of Padova, Via U. Bassi 58/B, Padova 35131, Italy; National Biodiversity Future Center, Piazza Marina 61, Palermo 90121, Italy

**Keywords:** anti-predator behavior, brain damage, climate change, heat stress, learning, mate choice

## Abstract

Heatwaves, increasingly common and intense due to climate change, are increasing mortality rates and disrupting vital functions. Recent research has begun exploring their impact on cognition. Since cognition underlies key fitness-related behaviors, such as foraging, predator avoidance, and mate choice, understanding the cognitive costs of heatwaves is crucial. Here, we investigate whether heatwaves impact cognition using male guppies (*Poecilia reticulata*) as a vertebrate model. We focused on males due to their behavioral consistency in cognitive tests and because they were previously observed to alter sexual behavior after a heatwave. Males were exposed to a 5-d experimental heatwave (32 °C) or control treatment (26 °C). The chosen temperatures are ecologically relevant for the species, fall within their natural habitat’s thermal range, and reflect extreme climatic events that are projected to become even more frequent and severe under future climate scenarios. Following treatment, all fish were tested at 26 °C for spatial memory and learning, mate choice, inhibitory control, and anti-predator responses. We also conducted histopathological evaluations of brain tissue to investigate potential central nervous system lesions. The results show that heatwave exposure declined maze solving efficiency, affected mate choice-related cognitive capacities, and led to suboptimal anti-predatory responses. No effects were observed on inhibitory control or habituation. Importantly, heatwave exposure induced morphological alterations in the central nervous system, potentially explaining the observed changes in cognitive performance. Our study provides a comprehensive evaluation of heatwave impacts on cognitive function, highlighting the need of investigating their subtle yet significant effects to fully understand how heatwaves influence fitness beyond survival.

## Teaser

Heatwaves are increasingly frequent and intense due to climate change, yet their effects on animal cognition remain poorly understood. We simulated heatwaves, mimicking those already occurring in the wild, to study their impact on cognitive traits in male guppies. The heatwave impacted learning, mate choice, and anti-predator responses, and also induced brain lesions, a potential cause of these behavioral changes. Understanding these effects is key to assessing how organisms may respond to climate change.

## Introduction

Heatwaves, defined as prolonged periods of abnormally hot weather, are increasingly common due to ongoing climate change (IPCC 2021). Once very rare, terrestrial heatwaves have increased in frequency since the 1950s and marine heatwaves have doubled since 1982, with projections indicating further escalation in frequency and intensity as global warming continues (IPCC 2021). These extreme warm events can be lethal for humans and wildlife alike (McKechnie and Wolf 2010; Xu et al. 2016; Piatt et al. 2020; IPCC 2021), but their impacts extend far beyond mortality. Growing evidence suggests that heatwaves can drive subtle yet profound changes in the physiology and behavior of organisms, potentially disrupting critical ecological processes (Buchholz et al. 2019; Stillman 2019). This can have broadly ranging implications for species distribution and abundance, by triggering range shifts, leading to changes in phenology, and causing population declines or expansions (Buckley and Huey 2016; Buchholz et al. 2019). Despite recently growing interest in this area (eg Cordonnier et al. 2023; Hemberger et al. 2023; Pilakouta et al. 2023; Barrett and Stein 2024; Grandela et al. 2024; Jawad et al. 2024), research investigating the behavioral consequences of heatwaves is still scarce, limiting our understanding of how species will cope with a warming climate.

A particularly understudied aspect is the potential impact of heatwaves on cognitive performance, a topic only recently highlighted by a handful of emerging studies (eg Blackburn et al. 2022; Gérard et al. 2022; Soravia et al. 2024). Cognition refers to the mental and neural processes through which individuals collect, process, retain, and use information from the environment (Shettleworth 2001). Cognition shapes behavior and behavioral flexibility, which in turn govern the initial responses to changing conditions and play a crucial role in allowing animals to adapt to novel environmental challenges (Sih et al. 2011; Wong and Candolin 2014; Beever et al. 2017; Ducatez et al. 2020; Lee and Thornton 2021). Cognition is vital for survival, and even minor cognitive impairments may have ramifications for multiple behavioral traits (Szabo et al. 2022). These impacts extend to essential behaviors such as foraging, mating, and predator avoidance, with potential consequences for both individual survival and the long-term viability of populations (Cunningham et al. 2021).

Heatwaves may affect animal behavior and the underlying cognitive processes in several interconnected ways. Heat stress can directly impair cognitive functions—leading to behavioral changes—through the direct effects of elevated temperatures on metabolism and neuronal activity (Rivest et al. 2019; Soravia et al. 2021). Heat stress can also compromise an organism’s physical condition, which can have cascading effects on behavior (Fuller et al. 1998; Abram et al. 2017). Additionally, animals may adjust their behavior to avoid heat stress, such as changing their activity patterns or engaging in heat dissipation behaviors (Cunningham et al. 2021). In all cases, heatwaves are expected to impact cognitive processes—including attention, learning, memory, and decision-making—which are crucial for essential behaviors like foraging, mate choice, and predator avoidance (Shettleworth 2001). Foraging, for example, relies on learning and memory to locate and recall the location of food sources (Szabo et al. 2022). In bumblebees (*Bombus terrestris*), heatwaves impair the ability to discriminate between food sources of varying quality, affecting the colony’s food supply (Gérard et al. 2022). Similarly, zebra finches (*Taeniopygia guttata*), show reduced cognitive performance during a heatwave, requiring more trials to obtain a food reward in a detour-reaching task (Danner et al. 2021).

Mate selection, which depends on learning, memory, and the flexible use of information (Bailey and Zuk 2009; Podos et al. 2009; Cummings and Ramsey 2015; Spezie et al. 2022), may also be affected by heatwaves (Coomes et al. 2019). When choosing a mate, individuals must locate potential partners, assess their quality (often in comparison to their own), and retain this information for future decision-making (Rosenthal 2017). Poor or suboptimal mate choice can have significant fitness consequences, by leading to reduced offspring number or quality. During heatwaves, female zebra finches are less able to discriminate between conspecific and heterospecific males, increasing the risk of hybridization (Coomes et al. 2019). Likewise, heatwaves could disrupt the ability of selecting between two or more conspecific mates, a process that is often based on small quality differences in multiple traits (Candolin 2019). They could also impact an individual’s sexual interest, ie it’s motivation to find or select a mate, since heat stress can cause alterations in the dopaminergic system, which regulates motivation and reward processing (Kim et al. 2013). Even subtle shifts in mate choice paradigms can drive evolutionary changes, altering the genetic diversity within populations and potentially influencing their adaptive potential (Rosenthal 2017). In addition, future mate preferences, which are often shaped through social learning (Ryan and Cummings 2013), may be affected by temporary cognitive impairments, with potential consequences for future mating decisions even long after the cognitive stressor has ceased.

Predator avoidance requires an ability to recognize predators, accurately assess the level of threat they pose, and make quick, adaptive, and context-dependent decisions to avoid being predated (Tollrian and Harvell 1999; Kelley and Magurran 2003; Ferrari et al. 2016). Heat-induced cognitive impairments can disrupt these critical processes, potentially increasing an organism’s vulnerability to predation. For example, during a heatwave, great tits (*Parus major*) exhibit a reduced response to conspecific alarm calls (Cordonnier et al. 2023), and snails (*Littoraria irrorate*) reduce predator avoidance behavior (Jawad et al. 2024). Likewise, under elevated temperature, damselfish (*Pomacentrus wardi*) show a decreased escape response, allowing predators to approach closer before fleeing (Allan et al. 2015).

Here, we determine the effects of a heatwave on the cognitive performance of guppies (*Poecilia reticulata*) across multiple behavioral tests. Our aim is to assess the impact of heatwaves on cognitive processes related to learning, memory and decision-making, particularly in the contexts of mate choice and anti-predator responses. The guppy is an ideal species for studying cognition, with a well-established line of research focusing on learning, problem solving, and inhibitory control (eg Shohet and Watt 2009; Kotrschal et al. 2013; Lucon-Xiccato and Bisazza 2017b; Santacà et al. 2019b; Mair et al. 2021; Vinogradov et al. 2024). Guppies, being ectotherms, cannot directly regulate their body or brain temperature (Angilletta 2009; Paaijmans et al. 2013), making them vulnerable to temperature changes. Moreover, guppies live in shallow freshwater habitats—where thermal conditions can fluctuate severely during extreme climatic events such as heatwaves (Ledger and Milner 2015; Woodward et al. 2016)—in regions where heatwave occurrence is an ecologically relevant problem (Stephenson et al. 2014 ; Angeles-Malaspina et al. 2018) .

We exposed male guppies to an experimental heatwave (32 °C) or a control temperature (26 °C) for 5 d. These temperatures are within the range of naturally occurring temperatures for the species (Reeve et al. 2014) and well below their CTmax (38 °C; Grinder et al. 2020). They align with a previous study on sublethal effects of heatwaves in adult guppies (Breedveld et al. 2023), and with the heatwave definition used by Sales et al. (Sales et al. 2018) , which describe a heatwave as temperatures that exceed the local average by 5 to 7 °C for at least 5 d. Following the treatment, we quantified cognitive performance across different behavioral tests. We focused on males because previous findings show that heatwaves affect male sexual behavior (Breedveld et al. 2023), and because males show greater behavioral persistence than females in the context of cognitive testing (Lucon-Xiccato and Bisazza 2014), facilitating the interpretation of behavioral test results. To assess the effects of heatwaves on cognition, in one group of males we tested memory and learning ability during repeated trials in a mate-search maze over 4 d. In another group of males, we tested performance in a battery of single-trial cognitive tasks, encompassing mate choice, inhibitory control, and anti-predator responses, over 2 d. Additionally, we performed a histopathological examination of the brain in a subset of males, to identify any morphological changes in the central nervous system that might explain the observed behavioral effects. Since heatwaves have previously been shown to affect male sexual behavior (Breckels and Neff 2013; Reeve et al. 2014; Breedveld et al. 2023), and other (non-thermal) abiotic stressors have been shown to affect cognitive performance in fish (Burns and Rodd 2008; Jacquin et al. 2020; Montalbano et al. 2022), we predict an overall negative impact of heatwaves on guppy cognition.

## Materials and methods

### Overview of the experimental design

**Fig. 1.**
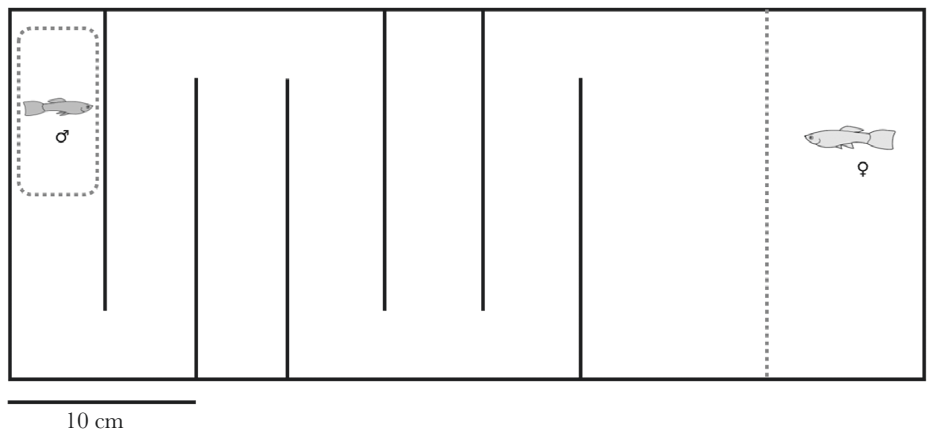
Schematic representation of the maze used to assess spatial learning. Solid lines represent opaque surfaces and dashed lines represent transparent surfaces. Males were initially placed in a transparent cylinder at the starting point and allowed to acclimate for 1 min. After acclimation, the cylinder was lifted, and the fish left free to explore the corridors of the maze. Upon reaching the final compartment (the one directly besides the reward), a transparent divider, behind which a virgin female was enclosed, was lifted, allowing the male access to the female (refer to the main text for more details).

**Fig. 2.**
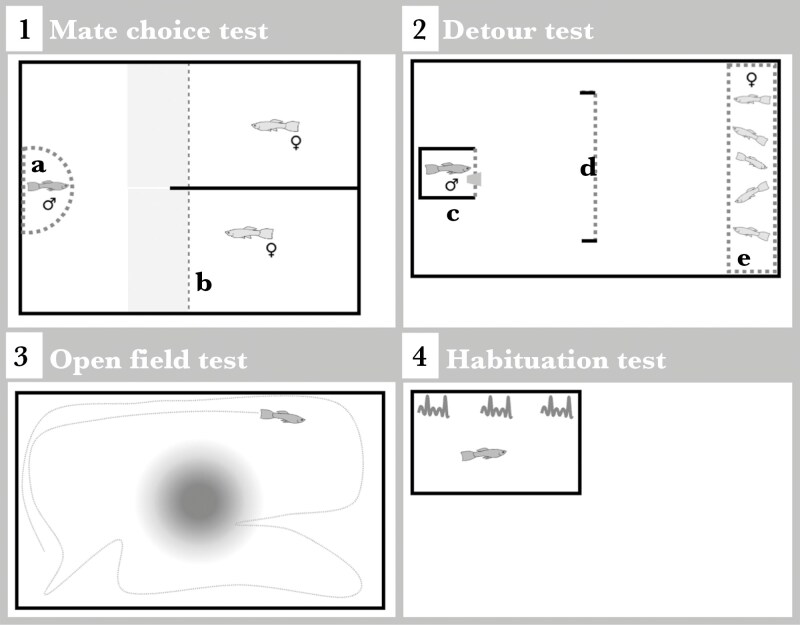
Schematic setup of the four behavioral tests used to assess cognitive abilities. Solid lines represent opaque surfaces and dashed lines represent transparent surfaces. 1) Mate choice test: This set up includes a Perspex semi-cylinder (a) used for male acclimation, and a glass divider (b) behind which two females were placed in separate compartments. The two female choice zones in the male’s compartment are shaded in gray. 2) Detour test: This set up includes a shelter with a window (c) used for male acclimation, an enclosure containing 5 stimulus females (d), and a barrier (e), obstructing the straight path between the shelter and the females. 3) Open field test: This set up includes free swimming activity and exploration analyses before and after exposure to a chemical alarm cue (represented as a circle). 4) Habituation test: This set up includes exposure to a repeated acoustic and vibrational stimulus (represented by wave symbols).

Our study aimed to investigate the effects of heatwaves on the cognitive performance of adult male guppies. Males used were descendent of wild-caught guppies from the Tacarigua River in Trinidad. Experimental males were maintained under standard lab conditions, as a stock population. Fish were housed in medium (50L) to large stock tanks (120L), at a temperature of 26 °C ± 1 °C, with a light:dark cycle of 12:12 h, and fed twice a day with a mix of brine shrimp (*Artemia salina* nauplii) and commercial dry food. Experimental males (7 ± 1 mo old) were randomly selected from stock tanks and randomly assigned to either one of two treatments: the heatwave group exposed to 32 °C (also referred to as HT), or the control group, maintained at 26 °C (also referred to as C). Behavioural tests were conducted immediately following the treatment. Since the maze test for assessing spatial memory and learning abilities required a 4-d protocol, we conducted this test in a first group of males (N = 90; Fig. 1). Another separate group of males (N = 115) was tested in four (non repeated) behavioral tests, which together spanned over 2 d (Fig. 2). Additionally, in a subset of males (N = 15), we performed histopathological evaluations of brain tissue, to investigate potential central nervous system lesions. These evaluations aimed to provide insight into any significant patterns observed in the behavioral results. To ensure objectivity, all behavioral tests and histopathological evaluations were conducted blind to the experimental treatment.

### Heatwave treatment

In the heatwave treatment, the temperature was set 6 °C above the control, reaching 32 °C for 5 d. On the first day, the temperature was gradually increased (1 °C/hour) from 26 °C to 32 °C, using aquarium heaters (100 W, NEWA Therm Pro), maintained at 32 °C for the next 5 d, and then gradually returned to room temperature (26 °C) on the seventh day. In the control treatment, the water temperature was kept constant at 26 °C using the same type of heaters. Water temperature in all tanks was accurately monitored using both a standard aquarium thermometer and temperature loggers (EnvLogger v2.4, ElectricBlueCRL). Due to logistical constraints, males were tested in blocks, with each block consisting of 5 individuals from the heatwave treatment and 5 from the control treatment. To facilitate tracking of individual subjects, each male was housed individually in a 2L tank within a recirculating water system (Tecniplast) during the behavioral trials.

### Spatial cognition and learning (maze test)

To assess whether exposure to a heatwave affects spatial cognition and learning in male guppies, we tested their performance in a maze using an associative learning protocol. In this test, the reward for successfully completing the maze was access to an unfamiliar virgin female. This setup has been previously validated in this species, as using a female as a reward mimics the natural scenario in which male guppies navigate through shallow water to find mates (Kotrschal et al. 2015; Lucon-Xiccato and Bisazza 2017a). Spatial ability is a particularly important cognitive skill for male guppies, as their reproductive success depends on mating with as many females as possible, driving strong selection for efficient movement in search of mates (Croft et al. 2003). The maze (Fig. 1) was set up in a 47 × 19 cm tank with opaque walls, filled with 10 cm of water, and with a thin layer of fine gravel covering the bottom. The corridors of the maze, each 5 cm wide with opaque walls, were designed to encourage guppies to navigate through the maze (Kellogg and Gavin 1960). Two corridors ended in a dead end to increase the difficulty and reduce biases caused by differences in swimming activity levels (Kotrschal et al. 2015). The maze was illuminated by two LED lights, and a video camera (Panasonic HC-V180) placed above the tank video recorded the trials.

Each fish was placed in the maze three times a day for four consecutive days in the morning (9:00 to 14:00 h). Between trials, it was returned to its individual tank for 1 h, and its three daily trials were performed at the same time each day. The first day served as an acclimation period to familiarize the fish with the setup and procedures, while the subsequent 3 d were used for actual testing. Thus, data for each male were collected over a total of 9 trials. At the start of each trial, the fish was placed in a transparent Perspex cylinder at the starting point of the maze and left to acclimate for 1 min. The cylinder was then lifted (manually), and once the fish left the starting corridor the time started. The fish was given a maximum of 10 min to complete the maze. The maze was considered successfully completed when the focal male reached the final compartment adjacent to the unfamiliar virgin female, at which point the female was released by lifting the transparent Perspex divider, allowing the male to interact with her for 2 min. Interactions were monitored but not scored, and no copulations were observed in this brief time. If the male failed to complete the maze within the 10 min, he was gently guided to the final compartment using a hand net and rewarded with access to the female as above, to facilitate positive associative learning, even during unsuccessful trials (Kotrschal et al. 2015). To avoid the risk that female identity systematically influenced male motivation in the task, we randomized females within and between males and treatments, using a total of 25 females. While virgin females were used more than once, we ensured that each female was used for both treatments and, moreover, that no male encountered the same female more than once.

For each trial, the following variables were visually extracted from the recorded videos: (a) *Solving probability*: whether the focal male completed the maze (ie autonomously reached the other end of the maze) within 10 min; (b) *Search time*: the time taken to reach the reward; and (c) *Number of errors*: the number of times the male entered a corridor with a dead end or swam backwards into at previous corridor (in the direction of the starting point). For the analyses, the maximum search time (600 s) was assigned for uncompleted maze trials.

### Mate choice

The mate choice test evaluated male mate choice. Given that mate choice is a cognitively demanding process involving the assessment and comparison of potential mates, we hypothesize that heat stress could impair the cognitive functions underlying these behaviors, leading to less strong or more inconsistent mate preferences. Mate choice tests are commonly used to assess male and female preferences and choosiness in this species (eg Gasparini et al. 2013; Kniel and Godin 2020), with association time serving as a reliable proxy for mating preference when fish are allowed to physically interact (Jeswiet and Godin 2011). One trait that influences male choice in this and other species is female body size, because it is a proxy for female quality, where larger females are usually more fecund (Herdman et al. 2004). In various studies, male guppies have indeed been shown to prefer the larger of two females (eg Herdman et al. 2004; Godin and Auld 2013), but not always (eg Jeswiet and Godin 2011; Kniel and Godin 2020; Schlupp 2021). Mate choice tests were conducted in the morning (8:00 to 13:00 h) on the first day following the treatment. The experimental tank (39 × 29 cm) followed a classical dichotomous mate choice apparatus design, consisting of a large compartment for the focal male and two smaller compartments for each of the stimulus females (Fig. 2.1). Two randomly selected females (total *N* = 30 females), differing in size (mean average differences between females in a test: mean ± SE = 5.8 ± 0.3 mm), were placed in their compartments and left to acclimate for 30 min before the test began. At the start of a trial, the male was placed in a transparent semi-cylinder to acclimate. After 5 min, the semi-cylinder was lifted, allowing the male to move freely for 25 min. During this time, the fish behavior was video recorded from above. The male could see and approach, but not physically interact with, the two females that were confined in the two dedicated compartments behind transparent glass barriers. The male’s compartment was virtually divided into one no-choice zone and two female choice zones, used to measure the time the male spent in each zone. To prevent the females from seeing each other and to avoid ambiguities in the male’s choice scoring, an opaque barrier separated the female compartments, extending slightly into the male’s compartment. This ensured that the male could only see one female when he entered a particular choice zone (and was not simply drifting from one zone to the other). Using the software BORIS (v.8.11.4), the following variables were obtained from the video recordings: (a) Time spent (in seconds) with each of the two females (which combined gave the total choice time), (b) Time spent in the no-choice area, and (c) The number of switches between the two females while choosing.

### Detour

The detour test was used to evaluate males’ inhibitory control, specifically their ability to navigate around (ie ‘detour’) a transparent barrier to reach a resource located behind it. Inhibitory control refers to the ability to suppress an inappropriate response, such as attempting to move directly through an obstacle blocking the direct path to the goal (Santacà et al. 2019a). The detour tests were conducted in the morning (8:00 to 11:00 h) of the second day following the heatwave. The experimental apparatus was designed following a set up previously used in this species (Santacà et al. 2019a). The tank (43×26.5 cm, filled to 4 cm in height) contained a shelter for the male, a transparent enclosure housing five stimulus females (the goal), and a transparent barrier positioned directly in the path between the shelter and the goal (Fig. 2.2). This transparent barrier forced the male to bypass it in order to reach the females, thus requiring the inhibition of the natural propensity to move directly toward them. The barrier was C-shaped, with two small opaque sides (following Lucon-Xiccato et al. 2017), designed to increase the difficulty of detouring it and to prevent accidental detouring when attempting to pass through the barrier. Before each trial, five females were randomly selected from a tank containing all the stimulus females (total *N* = 30) and released into the transparent enclosure. The behavioral trial was video recorded from above the tank. At the start of a trial, the male was placed in a shelter with a transparent window facing the center of the tank. The window featured a small circular opening (1 cm diameter), that was closed off with foam during acclimation. After 5 min, the foam was carefully removed allowing the fish to leave the shelter and move around the experimental tank for a maximum of 25 min. On the opposite end of the tank, the male could see and approach the females (the social reward) enclosed in the transparent container. Using the software BORIS the following variables were obtained: (a) Solved trial or not: whether the focal subject successfully detoured around the barrier to reach the goal (solved trial) or failed to do so (unsolved); (b) Incorrect versus correct trial: whether the focal subject reached the goal but touched the barrier at least once (incorrect trial), or successfully detoured around the barrier without touching it (correct trial); and (c) Time in front of the barrier: the time the focal subject spent attempting to pass through the barrier.

### *Exploration and anti-predator response* (open field test)

An open field test was performed to assess males’ activity and exploratory behavior in a novel environment, and their response to a simulated predator risk. The tests were conducted in the morning (11:00 to 12:30 h) on the second day after the end of the treatment. The arena consisted of a rectangular experimental tank (43×26.5 cm, filled to 4 cm in height) with opaque sides (Fig. 2.3). The male was released in the center of the tank and allowed to move around freely while being video recorded from above. After a 5-min acclimation period (not analyzed) there was a 10-min period of free swimming. After this, a chemical stimulus simulating the presence of a potential predator was added to the center of tank (final concentration: 0.05 ml/l, following Cattelan et al. 2020), and the fish was observed for another 10 min. The chemical stimulus (hereafter, ‘alarm cue’) was prepared from crushed conspecific skin – simulating the odor of injured conspecifics after a successful predator attack – following previously developed protocols (eg Evans et al. 2007; Cattelan et al. 2020). Briefly, 12 female guppies were randomly selected from stock populations and euthanized with an overdose of anesthetic (MS-222). The skin and underlying muscular tissue of each fish were homogenized with 2 ml of distilled water. After thorough mixing, the solution was centrifuged and filtered to produce a clear supernatant. The cue was prepared in advance and stored in aliquots in the freezer at −20 °C. From the video recordings, individuals were tracked using the software ToxTrac (version 2.98) and the following variables were recorded for each of the two periods (ie before and after the alarm cue was added): (a) Swimming velocity (mm/s); (b) Time spent freezing (seconds); and (c) Space use, measured as the number of different areas of the open field visited.

### Habituation

A habituation test was conducted to assess the male’s response to a repeated, non-harmful stimulus (acoustic and vibrational). This test evaluated the fish’s simple learning ability, specifically their capacity to learn to ignore (ie habituate to) a persistent yet inconsequential stimulus (Çevik 2014; Hong and Zha 2019). Habituation tests were performed in the afternoon (13:00 to 18:00 h) on the second day following the treatment. Each male was placed in a small container (18×11.5 cm) containing 0.5 L water, with opaque sides (Fig. 2.4). After a 5-min acclimation period (not scored), there was a 5-min period of free swimming (“before disturbance”), and then the fish was exposed for 10 min to a constant, standardized disturbance stimulus repeated every 7 s (85 repeats in total). The disturbance stimulus was generated by dropping a rod in a standardized way within a frame outside the tank, creating an acoustic and vibrational stimulus. The fish behavior was video recorded from above. Using the software ToxTrac, the following variables were extracted from the video: (a) Average swimming speed (mm/s); and (b) Time spent freezing (seconds). Freezing behavior is commonly used as an anti-predator response and reductions in freezing time would suggest habituation to the stimulus. The trial was divided into three different periods: 5 min before the stimulus exposure (“before disturbance”), the first 5 min after the onset of the disturbance stimulus (“short disturbance”), and the last 5 min during stimulus exposure (“long disturbance’). Males” responses to the stimulus were evaluated by comparing behavior before and after the disturbance (ie “before disturbance” vs “short disturbance”), while habituation to the stimulus was tested by comparing the “short disturbance” to the “long disturbance” period.

### Central nervous system histology

Ten randomly selected males (five from the heatwave group and five controls) were sacrificed 1 d after the end of the heatwave using an overdose of anesthetic (tricaine methanesulphonate, MS-222). All fish were fixed in 10% buffered formalin and embedded in paraffin. Five-micrometres thick sections of the cranial aspect of the fish, including hard and soft tissues, were obtained from each sample, dewaxed, processed, and stained with Haematoxylin-Eosin stain (H&E). Histopathological evaluation was performed on tangential sections through the eye and *tectum opticum* of each subject. The main histological features were evaluated by a board-certified veterinary pathologist (RV) and a veterinary pathologist (MG) as percentage of tissue involvement and scored as follows: - no findings (no tissue involvement); + minimal (up to 10% of involved tissue); ++ mild (up to 25% of involved tissue); +++ moderate (up to 50% of involved tissue); ++++ severe (up to 75% of involved tissue); +++++ massive (>75% of involved tissue). Although we focused on examining the brains of the fish sacrificed at the same time that the other fish underwent behavioral tests—to directly link histological results with behavioral outcomes—we also analyzed the brain tissue of an additional group of five fish that underwent heatwave exposure but were sacrificed 4 d later (rather than immediately after heatwave exposure, like the others). This “HT delayed group” was included to provide further context for interpreting our results.

### Statistical analyses

The effects of a heatwave on the cognitive performance of male guppies were investigated using both linear and generalized linear mixed-effects models (LMMs and GLMMs) with the package lme4 (Bates et al. 2015) in R version 1.4.0 (R Core Team. 2021). Since some males underwent treatment and were tested on the same days, we included the random effect of BLOCK (ie identifying the group of males undergoing treatment and behavioral trials simultaneously) in all models. When BLOCK was not significant, it was removed to simplify the models, which in some cases led us to use non mixed models (GLM and LM; Table S3). GLMMs were tested for overdispersion using the function dispersion_glmer from the package blmeco (Korner-Nievergelt et al. 2015) and corrected where necessary by adding an observation-level random effect (OLRE). The significance of fixed effects in LMMs was calculated from F statistics with the lmerTest package (Kuznetsova et al. 2017) and Satterthwaite’s approximation to calculate the denominator degrees of freedom. The significance of fixed effects in GLMMs was calculated from chisquare statistics, using Wald chi-square tests from the car package (Fox and Weisberg 2019). Post hoc comparisons were conducted using the emmeans and emtrends functions from the emmeans package (Lenth 2022), applying the Satterthwaite method for degrees of freedom approximation and Tukey’s adjustment for multiple testing. Mean ± SE are reported unless otherwise indicated. All the details on how the models were built are also summarized in the supplementary material (table S3).

#### Maze learning

 The performance of males in the repeated maze test was analyzed using a GLMM model. *Search time* (log transformed to improve normality; table S3) was analyzed with an LMM model, while *solving probability* and the *number of errors* were analyzed using GLMM models with binomial and Poisson family, respectively. All the models included TREATMENT (heatwave or control), TRIAL NUMBER (1 to 9), and their interaction as fixed factors. MALE ID was included as random effect to account for repeated measures of the same individual.

#### Mate choice

In a preliminary analysis, we found that, on average, males spent more choice time with the relatively smaller female than with the larger female (χ^2^ = 15.598, *P* < 0.001). However, males did not express a common preference for the smaller female, with 36 out of 99 males preferring the larger female. Therefore, as found in previous studies (eg Jeswiet and Godin 2011; Kniel and Godin 2020), the association between male preference and female body size was not straightforward, with other traits likely influencing male mate choice and causing inter-individual variation in male mating preferences (Schlupp 2021). Because of this variation in mating preferences, and because our main question was whether a heatwave affects the strength and consistency of the male mating preference and not on which female traits male choice is based, in our following analyses we classified the two females as ‘preferred’ female or ‘not preferred’ female, with the preferred female being the female with whom the male spent most of his choice time. The mean size difference between the two females did not significantly differ between treatments (t-test; t = 1.672, *P *= 0.098; mean ± SE, control: 5.4 ± 0.2 mm, heatwave: 6.3 ± 0.4 mm). However, to account for the potential influence of the female size difference on male preference, we included the size difference between the two females (in mm; FEMALE DIFF) as a covariate in all the models.

To investigate males’ overall investment in mate choice, ie their *sexual interest* (or motivation) (Table S3), we used a GLMM with a binomial distribution with the time spent in the choice area relative to time spent in the no-choice area (as a two-column response matrix using cbind) as the dependent variable, and the TREATMENT as fixed factor. To examine male mating preferences, we ran two models: preference strength and preference consistency. In the *preference strength* model, we investigated the differences in the relative association time with their preferred female versus their not preferred female. The model was built using a GLMM with binomial distribution, where the time males spent associating with their preferred female relative to the time associating with the not preferred female was the dependent variable (as a two-column response matrix using cbind), and TREATMENT the fixed factor. In the *preference consistency* model, we investigated the rate, per minute of choice time, of switches between the two females, where a higher rate indicates a less consistent choice, as the male switched more frequently between the two females. Switch rate (log transformed to improve normality; Table S3) was analyzed with an LMM model, with TREATMENT as a fixed factor. In an additional analysis, we examined whether heatwaves affect the influence of female size on male choosiness. This was done by testing the interaction between TREATMENT and the female size difference (FEMALE DIFF) in the three beforementioned mate choice models. As this analysis explores the underlying mechanisms of heatwave effects on male choosiness, which goes beyond our main question of whether heatwaves influence male choice, we have presented these results in the supplementary material (S.5).

#### Detour test

All models for the detour test included TREATMENT as a fixed factor. *Solved trial or not* and *correct trial or not* were analyzed using GLMM with a binomial distribution. *Time in front of the barrier* was analyzed using an LMM.

#### Exploration and anti-predator response (Open field test)

All models analyzing behavior in the open field included TREATMENT, STIMULUS (*before* or *after* the alarm cue), and their interaction as fixed factors, and MALE ID as random effect to account for individual repeated measures. *Average swimming speed* was analyzed using an LMM. The ratio of time spent freezing relative to time not freezing (*freezing time*, Table S3), as well as the ratio of space used relative to space not used (*space use*), were analyzed using GLMMs with a binomial distribution.

#### Habituation test

All models analyzing behavior in the habituation test included TREATMENT, STIMULUS (*before disturbance*, *short disturbance*, or *long disturbance*), and their interaction as fixed factors, and MALE ID as a random effect to account for individual repeated measures. *Average swimming speed* (log-transformed to improve normality) was analyzed using LMMs, and the *ratio of time spent freezing*, relative to time not freezing (as a two-column response matrix) was analyzed using a GLMMs with a binomial distribution.

#### Sample sizes

The two groups of males we used initially consisted of 90 and 115 males, but 8 and 12 males died, respectively, during the experiment. For the analyses, some males were excluded for technical reasons, such as poor video quality (ie the video quality was insufficient for reliable tracking of the fish). The final sample sizes used in each of the statistical models are clearly stated in the results and in Table S3.

## Results

### Maze test

**Fig. 3.**
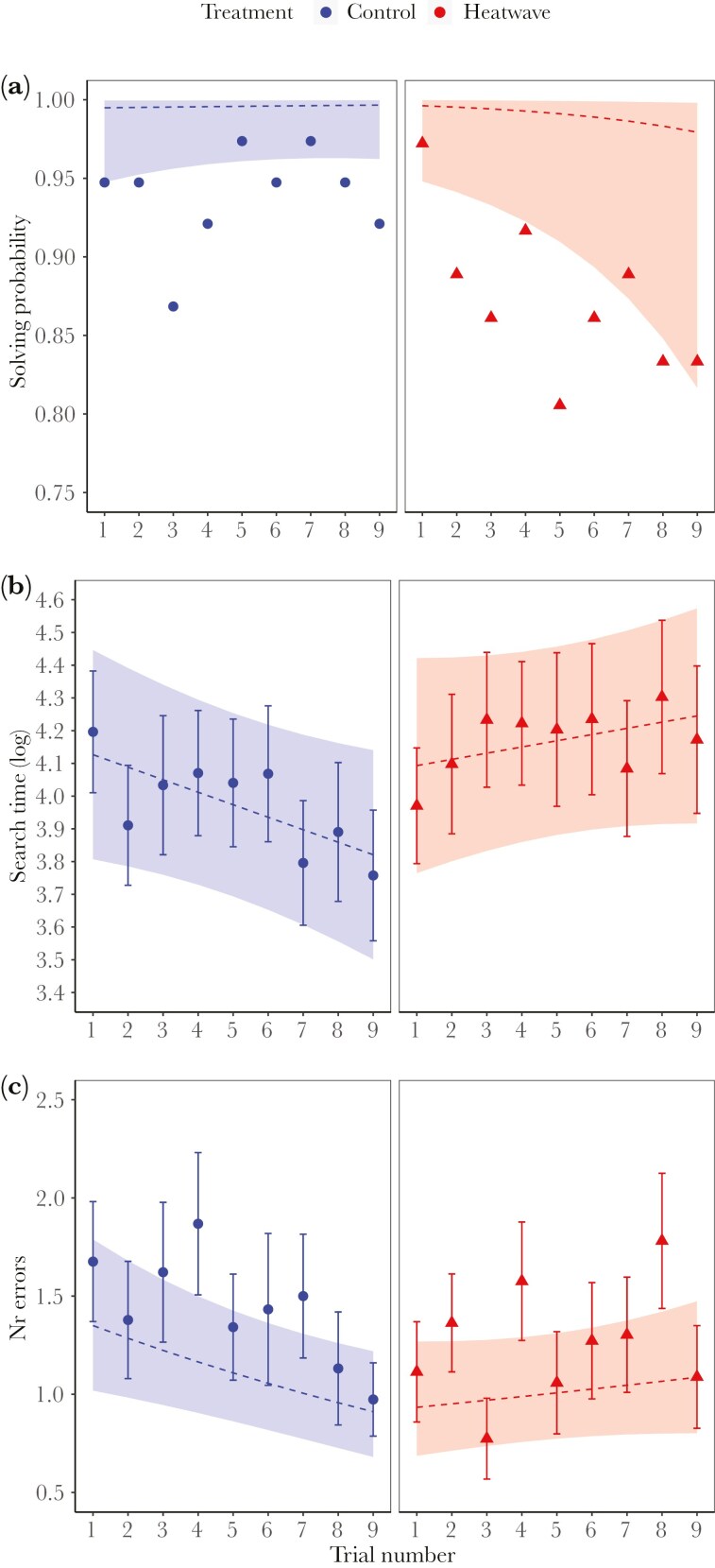
Results from the maze test after exposure to a heatwave (red triangles; N = 36) or control treatment (blue circles; N = 38). Performance during the 9 repeated trials (on the X axis) in A) Solving probability (plotted as the proportion of males solving each trial), B) Search time (as mean ± SE per trial), and C) Number of errors (as mean ± SE per trial). The models’ predicted slopes (smoothed estimates based on the fitted model; dashed line) and confidence intervals (SE of the predicted values; shade around the slope) are also shown.

We obtained data from 74 adult male guppies that performed all 9 trials of the repeated maze test, 36 heatwave and 38 control males. The overall solving probability was high; control and heatwave males completed 94% and 87% of all maze trials within 10 min, respectively. Solving probability (Fig. 3A) remained consistently high in the control group (post-hoc: z = 0.504, *P* = 0.614) while it decreased with trial number in the heatwave group (post-hoc: z = −2.229, P = 0.026), though the difference between the slopes was marginally non-significant (Table 1; TREATMENT × TRIAL NUMBER). As expected in a learning curve, the overall efficiency to navigate the maze improved over time, however this improvement was observed only in control males, not in heatwave males, as indicated by the significant interactions between treatment and trial number (Table 1). Search time (Fig. 3B) decreased with trial number in the control (post-hoc: t = −1.947, *P* = 0.052) but not in the heatwave group (post-hoc: t = 0.940, *P* = 0.348), indicating that males in the control group improved their ability to navigate the maze (shorter search time), while their heatwave counterparts did not. In line with this, number of errors (Fig. 3C) decreased in the control (z = −2.810, *P* = 0.005), but not in the heatwave group (post-hoc: z = 0.958, *P* = 0.337), though it must be noted that the number of errors made was generally low (mean = 1.3 ± 0.1, range: 0 to 12).

**Table 1.** **Results from the maze test.** Performance in a repeated maze test by male guppies recently subjected to a heatwave. The table presents the results from (G)LMM models testing each variable in response to the TREATMENT (control or heatwave), the TRIAL NUMBER (1 to 9), and their interaction. Details of the models used are reported in the main text. F statistics for LMM’s are given with their corresponding denominator degrees of freedom (dff) and χ2 statistics for GLMM’s are underlined. Significant effects are printed in bold.

	TREATMENT			TRIAL NUMBER			TREATMENT × TRIAL NUMBER			
	estimate (se)	*F*(ddf)/*χ2*	*p*	estimate (se)	*F*(ddf)/*χ*^*2*^	*p*	estimate (se)	*F*(ddf)/*χ*^*2*^	*p*	Figure
**Heatwave effects on spatial learning and memory**										
Solving prob.	0.560(1.191)	*0.81*	0.368	0.051(0.101)	*1.63*	0.202	−0.264(0.140)	*3.58*	0.058	Fig. 3A
Search time	−0.091(0.248)	0.13 (152)	0.716	−0.038(0.020)	0.55(590)	0.460	0.057(0.028)	4.13(590)	**0.043**	Fig. 3B
Nr of errors	−0.455(0.257)	*0.25*	0.620	−0.055(0.025)	*1.33*	0.249	0.074(0.037)	*4.04*	**0.045**	Fig. 3C

#### Mate choice

**Fig. 4.**
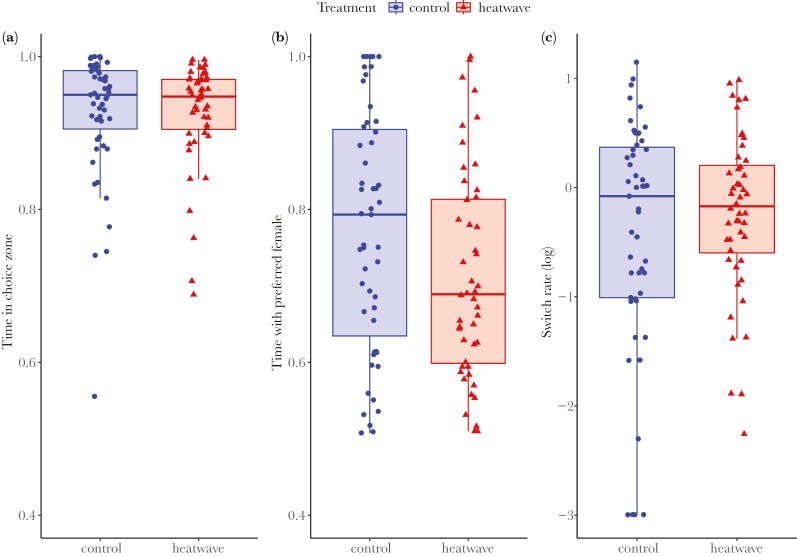
Results from the mate choice test after exposure to a heatwave (red triangles; N = 48) or control treatment (blue circles; N = 51). The proportion of time males spent A) in the choice zone (relative to the no choice zone), B) associating with their preferred female (relative to their non-preferred female), and C) the rate of switches (per minute of choice time) between the two females. Boxes represent the interquartile range (IQR), with whiskers extending to 1.5 times the IQR from the first and third quartiles.

We obtained data from 99 males in total, 48 heatwave and 51 control males. Although not significant, heatwave males tended to spend less time in the choice area relative to the no-choice area compared to control males (TREATMENT: χ^2^ = 3.352, *P* = 0.067; Fig. 4A), suggesting an overall decrease in sexual interest towards females. When analyzing male mating preferences, males’ proportion of time with their preferred female versus time with their non-preferred female, ie the strength in male mate choice, was affected by treatment (TREATMENT: χ^2^ = 6.980, *P* = 0.008; Fig. 4B). Specifically, heatwave males spent a lower proportion of their time (~14% lower) with their preferred female than control males, who spent ~88% of their time with their preferred female (Table S.4), indicating a weaker preference strength in heatwave males. The males’ rate of switching between the two females was higher in heatwave males (expected switch rate ≈ 0.8 switches per minute; Table S.4) than in control males (~ 0.6 switches per minute; TREATMENT: χ^2^ = 6.681, *P* = 0.010; Fig. 4C), pointing to a lower preference consistency in the former.

Female size difference was included as a covariate in the models to account for the influence of possible differences across trials, and, as expected, positively affected males’ sexual interest (estimate ± SE = 0.113 ± 0.055, χ^2^ = 4.167, P = 0.041), preference strength (trend; estimate ± SE = 0.124 ± 0.066, χ^2^ = 3.516, *P* = 0.061), and preference consistency (estimate ± SE = −0.0846 ± 0.034, χ^2^ = 6.237, *P* = 0.013), with males exposed to a pair of females differing more in size being more sexually interested and exhibiting a stronger, more consistent preference. For exploration of treatment dependent effects of female size difference on male choosiness, please see the supplementary material (S.5).

#### Detour test

We obtained data from 93 males in total, 46 heatwave and 47 control males. 87% of the heatwave males and 85% of control males successfully solved the detour task, and 9 and 5 males, respectively, detoured the barrier without ever touching it. Treatment did not affect whether the males were able to solve the trial (χ^2^ = 0.066, *P* = 0.797), whether the trial was correct or incorrect (χ^2^ = 1.465, *P* = 0.226), or the amount of time males spent in front of the barrier (*F*_1,91_ = 1.328, *P* = 0.252; Figure S1).

#### Anti-predator response

**Fig. 5.**
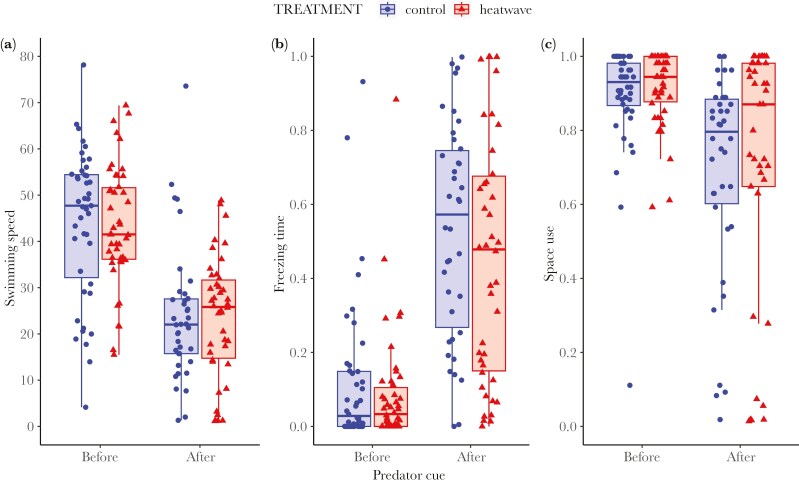
Results from the open field test after exposure to a heatwave (red triangles; N = 46) or control treatment (blue circles; N = 44). The three panels display behavior before (left, x-axis) versus after (right, x-axis) the presence of a predator cue (a conspecific alarm substance that signals a successful predator attack). A) Average swimming speed (mm/s), B) proportion of time spent freezing, and C) space use, that is the proportion of areas visited. Boxes represent the interquartile range (IQR), with whiskers extending to 1.5 times the IQR from the first and third quartiles.

We obtained data from 90 males in total, 46 heatwave and 44 control males. Males significantly adjusted their behavior in the presence of the alarm cue, in comparison to before the cue was present. Specifically, males reduced their swimming speed (*F*_1,83.1_ = 166.967, *P* < 0.001; Fig. 5A), increased their freezing time (χ^2^ = 120.144, *P* < 0.001; Fig. 5B), and reduced their space use (χ^2^ = 597.542, *P* < 0.001; Fig. 5C; see also Table S.4). These responses were independent of treatment (*P* > 0.4) for all but one variable, space use, for which there was a significant interaction between treatment and the presence of the stimulus (χ^2^ = 11.163, *P* < 0.001; Fig. 5C). While all males reduced the areas visited in response to the alarm cue, the reduction in space use was less pronounced in males from the heatwave group, with heatwave and control males showing a ~16% and ~18% reduction in space use, respectively (Table S.4).

#### Habituation test

**Fig. 6.**
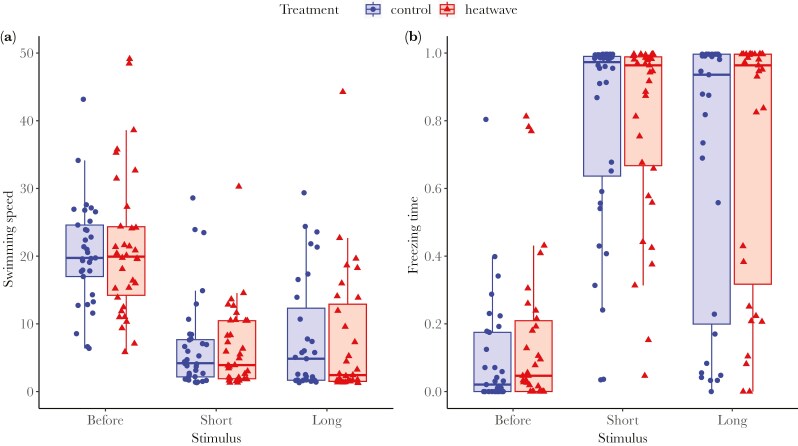
Results from the habituation test after exposure to a heatwave (red triangles; N = 35) or control treatment (blue circles; N = 36). Both panels display data for three time periods along the x-axis: “before disturbance” (left), “short disturbance” (center), and “long disturbance” (right). A) Average swimming speed (mm/s), and B) proportion of time spent freezing. Boxes represent the interquartile range (IQR), with whiskers extending to 1.5 times the IQR from the first and third quartiles.

We obtained data from 71 males in total, 35 heatwave and 36 control males. Swimming speed (Fig. 6A) was significantly affected by STIMULUS (*F*_2,128.7_ = 119.542, *P* < 0.001), but not by TREATMENT (*F*_1,68.8_ = 0.036, *P* = 0.849) or the interaction (TREATMENT × STIMULUS: *F*_2,128.7_ = 0.176, *P* = 0.839). Post hoc comparisons revealed that males from both groups significantly and similarly reduced swimming speed from before disturbance to short disturbance (C: estimate ± SE = 1.450 ± 0.155, t ratio = 9.381, *P* < 0.001; HT: estimate ± SE = 1.455 ± 0.154, t ratio = 9.460, *P* < 0.001), and maintained low swimming speed from short disturbance to long disturbance (C: estimate ± SE = 0.010 ± 0.158, t ratio = 0.061, *P* = 1; HT: estimate ± SE = 0.124 ± 0.159, t ratio = 0.784, *P* = 0.970). This indicates, respectively, that males from both groups responded to the stimulus by decreasing swimming speed, and that they did not habituate to the stimulus in terms of swimming speed. The lack of habituation is confirmed by the significant difference in swimming speed between the before disturbance and long disturbance period (C: estimate ± SE = 1.460 ± 0.162, t ratio = 9.037, *P* < 0.001; HT: estimate ± SE = 1.579 ± 0.159, t ratio = 9.919, *P* < 0.001).

Freezing time (Fig. 6B) was significantly affected by STIMULUS (χ^2^ = 325.367, *P* < 0.001), but not by TREATMENT (χ^2^ = 0.319, *P* = 0.572) or the interaction (TREATMENT × STIMULUS: χ^2^ = 1.892, *P* = 0.388). Post hoc comparisons revealed that freezing rates for both groups increased significantly from before disturbance to short disturbance (C: estimate ± SE = −7.321 ± 0.576, z ratio = −12.708, *P* < 0.001; HT: estimate ± SE = −6.226 ± 0.558, z ratio = −11.164, *P* < 0.001) and did not change significantly from short disturbance to long disturbance (C: estimate ± SE = 0.794 ± 0.558, z ratio = 1.419, *P* = 0.715; HT: estimate ± SE = 0.323 ± 0.562, z ratio = 0.594, *P* = 0.992). This indicates, respectively, that males from both groups responded to the stimulus by increased freezing, and that they did not habituate to the stimulus in terms of freezing. The lack of habituation is confirmed by the significant difference in freezing rate between the before disturbance and long disturbance period (C: estimate ± SE = −6.529 ± 0.603, t ratio = −10.827, *P* < 0.001; HT: estimate ± SE = −5.892 ± 0.583, t ratio = −10.113, *P* < 0.001).

### Central nervous system histology

**Fig. 7.**
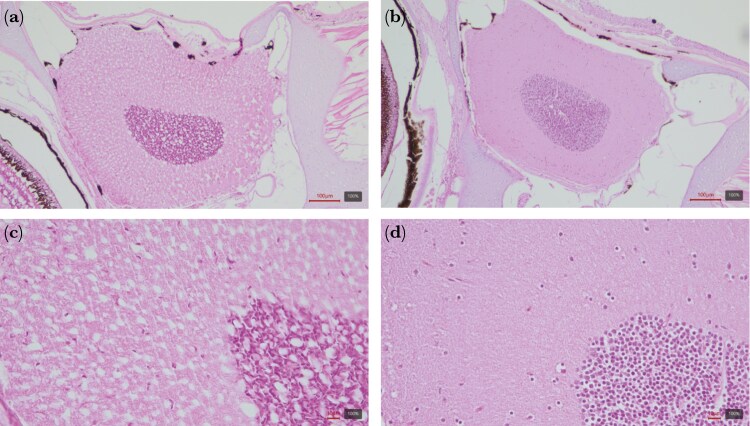
Histological section of the brain showing evident diffuse vacuolation in the white and gray matter following a heatwave (left: A, C; FMHDI 4) along with a control male (right: B, D; CTRL 3). H&E staining: A and B 10X ob. magnification; C and D 40X ob. magnification.

The major histological findings in all the heatwave subjects, with varying severity, were characterized by diffuse perineuronal edema and vacuolization both affecting the white and the gray matter of the brain. The results are reported in Table 2 and shown in Fig. 7. At high magnification (63X), the vacuoles were characterized by lightly eosinophilic to optically empty content, often displacing neuronal nuclei, suggesting possible intracellular and extracellular edematous changes with poorly proteinaceous, watery accumulation. Notably, no inflammation or necrosis was observed in any of the tested subjects. Fish in the control group did not show any of these changes and were within normal morphological limits. In addition, those fish that were sacrificed 4 d after the end of the heatwave (HT delayed group) did not show brain vacuolization or exhibited only very mild residual perineuronal edema. The histological results of the HT delayed group are detailed in supplementary material (S.2).

**Table 2.** Histological features of five males after heatwave treatment (FMHDI 1-5) compared to five males from the control (CTRL1-5). Classification: - no findings (no tissue involvement); + minimal (≤ 10% of tissue involved); ++ mild (≤ 25%); +++ moderate (≤ 50%); ++++ severe (≤ 75%); +++++ massive (>75%).

Treatment	Fish ID	Gliosis	Neuronal vacuolation	Perineuronal edema	Vacuolation/spongiosis of white matter
HEATWAVE	FMHDI 1	-	++++	++++	+++++
	FMHDI 2	-	+++	+++	+++
	FMHDI 3	-	+++	+++	+++
	FMHDI 4	-	++	+++	+++
	FMHDI 5	-	++	++	++++
CONTROL	CTRL 1	-	-	-	-
	CTRL 2	-	-	-	-
	CTRL 3	-	-	-	-
	CTRL 4	-	-	-	-
	CTRL 5	-	-	-	-

## Discussion

A growing body of research shows that heatwaves can induce changes in key fitness-related behaviors—including reproductive behavior (Breedveld et al. 2023; Pilakouta et al. 2023; Barrett and Stein 2024; Grandela et al. 2024), foraging behavior (Funghi et al. 2019; Hemberger, Rosenberger and Williams 2023), and anti-predator behavior (Cordonnier et al. 2023; Jawad et al. 2024; Breedveld et al. 2025)—thereby posing a significant threat to animal health and survival. In line with this, our findings in male guppies show that heatwaves alter behavior and cognition. Exposure to high temperature for 5 d under laboratory conditions—simulating an ecologically relevant heatwave—negatively affected cognitive performance across multiple tasks. Specifically, the heatwave resulted in (i) lower performance in a maze test; (ii) reduced sexual interest; (iii) weakened sexual preferences; and (iv) altered anti-predator responses. These behavioral changes reflect the impact of heatwaves on various underlying cognitive processes, including learning, memory, attention, and decision making, which are crucial for key activities such as locating essential resources, finding and choosing mates, and recognizing and avoiding predators (Shettleworth 2001; Szabo et al. 2022). Our findings also reveal that heatwaves induce changes in the central nervous system, that is, the neural substrate underlying cognition. These neurological alterations provide a potential morphological explanation for the observed changes in cognitive performance and behavior. Through subtle yet widespread effects on cognition and subsequent behavior, heatwaves can significantly impact an animal’s key fitness-related traits, such as reproduction and anti-predator behavior. This may ultimately jeopardize survival and impact sexual selection mechanisms—in line with recent findings in this species on other aspects of sexual selection (Breedveld et al. 2023)—potentially affecting the long-term viability of populations (Shettleworth 2001).

### Heatwave effects on cognitive performance

Heatwave exposure affected the maze solving efficiency of guppies, as has been previously shown for other stressors (eg predation risk; Burns and Rodd 2008). As expected in this type of spatial task, performance in the control fish followed a learning curve, that is, the fish became faster at reaching the reward (a virgin female) and showed reduced error rates over time (Kotrschal et al. 2015; Lucon-Xiccato and Bisazza 2017a). Heatwave-exposed males, however, showed no improvement in their efficiency of solving the maze, as they did not become faster in reaching the reward, and there was no reduction in their error rate. In addition, solving probability, which was overall high as expected, declined across successive trials in heatwave-exposed males, while it remained consistently high in the control group, though the difference between the slopes was marginally non-significant. The lack of improvement over time points to potential effects of heatwaves on spatial memory and learning ability, by which heatwaves could disrupt critical behaviors such as finding food, shelter, social companions, or mates (Szabo et al. 2022). An alternative, but not mutually exclusive, explanation for this result is that heatwave exposure led to a decrease in the fish’s general activity or in their motivation to reach the female at the end of the maze, possibly due to a reduced interest in females. This interpretation aligns with previous findings showing reduced sexual activity in heatwave-exposed male guppies (Breedveld et al. 2023) and with the results from the mate choice test in the current study, where heatwave males spent less time in the choice area, suggesting reduced sexual interest or motivation, compared to control males.

The mate choice test also revealed that the mating preference of heatwave-exposed males was less pronounced. Compared to control males, heatwave-exposed males showed weakened choosiness – they spent a lower ratio of time with their preferred female and were less consistent in their choice, exhibiting a higher rate of switching between the females. This indicates that heatwaves cause males not only to be less interested in females (ie less motivated to mate), but also alter the mate choice decision process. Although less studied relative to female mate choice, male mate choice is crucial for reproductive success and has far-reaching evolutionary implications (Schlupp 2021). Males in this and similar species have been shown to exhibit mate choice both at pre- and post-mating stages (eg Schlupp 2018). These choices, while sometimes subtle in their expression, can be frequent and involve complex decision-making processes, influenced by subtle differences among potential mates or by the social context (Rosenthal 2017). For example, male guppies prefer females surrounded by less attractive competitors and this depends on their own level of attractiveness (Gasparini et al. 2013). The weakened choosiness and reduced choice consistency in heatwave-exposed fish suggest a reduced ability in these decision-making processes. This may result from a decrease in their ability to assess female quality based on size, as suggested by the lower responsiveness of heatwave-exposed fish, compared to control fish, to the magnitude of female size differences (see supplementary material S.5). Heatwave-driven reductions in males’ ability or motivation to find, approach, and select mates can have clear negative implications for their reproductive success. In guppies, these effects may be further exacerbated by changes in sexual behavior. Male guppies exposed to a heatwave altered their mating tactics, shifting from courtship to forced copulation (Breedveld et al. 2023). While courtship is energetically costly, it typically results in higher sperm transfer compared to forced copulations (Pilastro and Bisazza 1999). Our current findings, combined with these behavioral changes, underscore the profound effects heatwaves can have on reproduction, and their potential repercussions on sexual selection dynamics.

There was a heatwave effect on how males responded to a predator threat in the open field. Following the introduction of a conspecific alarm cue, signaling the presence of a predator, all the fish reduced their swimming speed and space use while increasing their freezing rate, indicating that both heatwave and control fish perceived and reacted to the cue. However, the space use reduction differed between heatwave-exposed and control males, since heatwave males showed a less pronounced decrease in space use. These findings suggest that heatwaves do not necessarily interfere with the perception of a predator stimulus, but do alter how male guppies process and use the information about a potential predator. This aligns with our recent findings in juvenile guppies, where individuals exposed to a similar heatwave treatment exhibited reduced antipredator responses compared to controls (Breedveld et al. 2025). However, we cannot entirely rule out that high temperatures may have affected cue perception, since the functioning of sensory receptors can be temperature-dependent (eg olfactory receptors; Martin et al. 2011). Temperature could thereby alter the quality of information acquired from the environment, potentially triggering a cascade of effects on the subsequent behavioral response. Additionally, disruptions in the acquisition of information may act synergistically with changes in central nervous system information processing. Heatwave-induced disruptions in anti-predator responses could increase prey detection by predators, thereby elevating predation risk (Cordonnier et al. 2023). This could potentially alter predator-prey interactions and trigger cascading effects on communities of interacting organisms (Pintanel et al. 2021).

We also tested inhibitory control, an important component of decision-making that allows individuals to respond appropriately to stimuli by suppressing impulsive or maladaptive actions. The detour test, also known as the barrier task, is a well-established test for evaluating inhibitory control in the guppy and other animals (Lucon-Xiccato et al. 2017; Lucon-Xiccato 2024). In guppies, inhibitory control – the cognitive process that allows an individual to inhibit an inappropriate response, such as bypassing an obstacle to reach a resource behind it – has been extensively demonstrated (eg Santacà et al. 2019a). Inhibitory control can be context dependent, varying with factors such as the social environment (Lucon-Xiccato et al. 2022), and is predicted to be sensitive to environmental conditions (Prentice et al. 2023), including rearing temperature (Vinogradov et al. 2024). In birds, temperature has been shown to affect inhibitory behavior (Danner et al. 2021; Soravia et al. 2023). Interestingly, our study found no effects of heatwaves on inhibitory control performance of guppies. This suggests that certain cognitive processes may be more resilient to environmental stressors than others (Maille and Schradin 2017). However, alternative methods of measuring inhibitory control, such as a repeated cylinder task (Santacà et al. 2019a), might reveal heatwave effects on individuals’ ability to inhibit inappropriate responses that were not captured by our current approach. Future research building on these findings will help clarify whether inhibitory control is truly unaffected by heat stress or if specific conditions might reveal subtle effects.

Finally, we tested habituation to a repeated, non-harmful stimulus (acoustic and vibrational), to assess the fish’s ability to learn to disregard a persistent yet inconsequential stimulus (Çevik 2014; Hong and Zha 2019). All fish, regardless of treatment, responded similarly by reducing swimming speed and increasing freezing behavior, suggesting that heatwaves did not alter the fish’s perception of the stimulus. Interestingly, while heatwave exposure altered responses to the alarm cue in the open field test, it did not clearly affect the fish’s response to the habituation stimulus. Although our results hint at a subtle behavioral shift after prolonged stimulus exposure (Fig. 6; long stimulus vs short stimulus), we found no significant evidence for habituation or for differences in habituation between treatments within the timeframe of our test. We therefore cannot exclude the possibility that heatwave effects on habituation might emerge over longer exposure periods. This limitation suggests a need for future studies with extended observation periods, or different experimental setups to better understand the potential effects of heatwaves on this specific learning process.

### Heatwave effects in the central nervous system

Histological analysis revealed that heatwave-exposed guppies exhibited severe vacuolization throughout the central nervous system, affecting both gray and white matter, along with perineuronal edema (localized swelling around neurons). These lesions were completely absent in control fish. Vacuolization, also known as spongiform change, refers to the formation of small, fluid-filled spaces (vacuoles) within tissues, giving them a sponge-like appearance. Although the vacuolization observed in the neurons of heatwave-exposed males was classified as severe, it was not accompanied by signs of extensive neuronal damage (eg central chromatolysis) or surrounding inflammation (satellitosis and gliosis). The content of the vacuoles was morphologically compatible with water accumulation (intracellular and extracellular edema) consistent with heat-induced vacuolization previously reported in fish (Madeira et al. 2014), laboratory animals (El-Borai et al. 2023), and humans (Bazille et al. 2005). Given the role of the central nervous system in cognitive function and behavior, it is plausible that these structural changes contributed to the behavioral alterations observed in heatwave-exposed fish. Importantly, these lesions appeared to be transient, as the brain of heatwave-delayed fish showed either complete recovery or only mild residual alteration 4 d after exposure ended (see supplementary material S.2). Therefore, our results suggest that heatwave exposure induces significant but potentially reversible brain lesions in guppies. This rapid recovery of the brain suggests a high degree of neural plasticity, typical of fish’s brain (Lucini et al. 2018), which may help mitigate the impact of abrupt temperature changes in the wild as it happens during a heatwave (Ghaddar et al. 2021). Future research should explore whether behavioral recovery follows a trajectory similar to that of the brain, and examine if longer or repeated heatwave exposures have more severe impacts on brain functions.

## Conclusion

Our findings provide evidence that heatwaves affect cognitive performance in a freshwater fish, affecting key behaviors related to mating success and predator avoidance. This expands the growing body of research demonstrating the impact of heatwaves on animal cognition (eg (Danner et al. 2021; Blackburn et al. 2022; Gérard et al. 2022; Soravia et al. 2023). By disrupting cognitively demanding behaviors essential for reproduction and survival, heatwaves may cause cryptic yet profound effects at individual and population levels. Thereby, their effects can extend beyond lethality, and have potential far-reaching ecological impacts (Gurgel et al. 2020; Parratt et al. 2021; Starko et al. 2023). Moreover, by impairing anti-predator responses, heatwaves may not only hinder individual survival but also disrupt predator-prey dynamics, potentially triggering cascading effects on ecosystems (Pintanel et al. 2021). Importantly, we have linked these behavioral effects to significant, albeit transient, lesions in the central nervous system. This connection paves the way for future studies integrating behavioral assessments with neurophysiological and histological investigations to further elucidate the cognitive and neural impacts of heatwaves. Future research should also investigate how cognitive impairments develop during heatwaves, rather than only after exposure. Testing individuals mid-heatwave, for example, could provide insights into the trajectory and severity of the behavioral effects, or reveal additional short-term cognitive and physiological effects that may not have persisted post-exposure. A deeper understanding of these subtle yet ecologically significant sublethal effects is essential for predicting population resilience in an increasingly warming and unpredictable world.

## Supplementary Material

araf061_suppl_Supplementary_Material

## Data Availability

Analyses reported in this article can be reproduced using the data provided by Breedveld et al. 2025 (https://doi.org/10.5061/dryad.h44j0zpxc).
