# Supplementary material for: Too hot to reason? Experimental heatwaves affect cognitive traits in male guppies
Source: Behav Ecol. 2025 May 30;36(4):araf061. doi: 10.1093/beheco/araf061 (PMC12203089; doi:10.1093/beheco/araf061)
Supplement: araf061_suppl_Supplementary_Material [file araf061_suppl_supplementary_material.pdf]

Supporting information for:

Too hot to reason? Experimental heatwaves affect cognitive traits in male guppies

Merel C. Breedveld, Luna Dudine, Samuele Padovan, Marta Giacomazzo, Ranieri Verin, Clelia Gasparini

---

S.1 Detour test; plot for time in front of the barrier

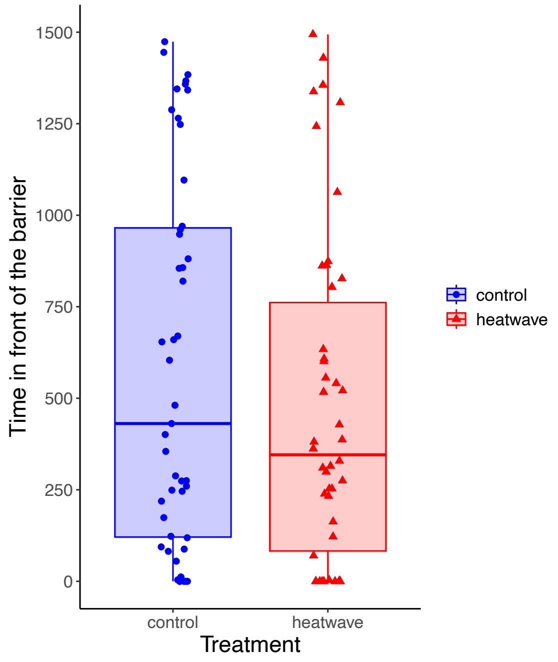

Figure S.1 Results of the detour test performed in male guppies following heatwave exposure (red triangles; N = 46) or control treatment (blue circles; N = 47), showing the amount of time males spent in front of the barrier. Boxes represent the interquartile range (IQR), with whiskers extending to 1.5 times the IQR from the first and third quartiles.

S.2 CNS histology of the Heatwave-delayed group

Histological results from an additional group of five fish that were exposed to a heatwave and sacrificed four days after the end of the exposure (HT delayed group). In contrast to fish examined directly after heatwave exposure (see main text), fish from the HT delayed group did not show brain vacuolization or, at most, exhibited only very mild residual perineuronal edema. Four out of five fish did show a mild presence of oligodendrocytes in the grey matter, interpreted as proliferation and activation of glial cells (gliosis) in response to non-specific brain injury, possibly induced by the heatwave.

Treatment	Fish ID	Gliosis	Neuronal vacuolation	Perineuronal edema	Vacuolation/spongiosis of white matter
HEATWAVE DELAYED GROUP	FMHDE 1	+	-	+	-
	FMHDE 2	-	-	+	-
	FMHDE 3	+	-	-	-
	FMHDE 4	+	-	-	-
	FMHDE 5	+	-	+	-

Table S.1 Histological features of five males four days after the end of heatwave exposure (FMHDE 1-5). Classification: - no findings (no tissue involvement); + minimal (≤ 10% of tissue involved); ++ mild (≤ 25%); +++ moderate (≤ 50%); ++++ severe (≤ 75%); +++++ massive (>75%).

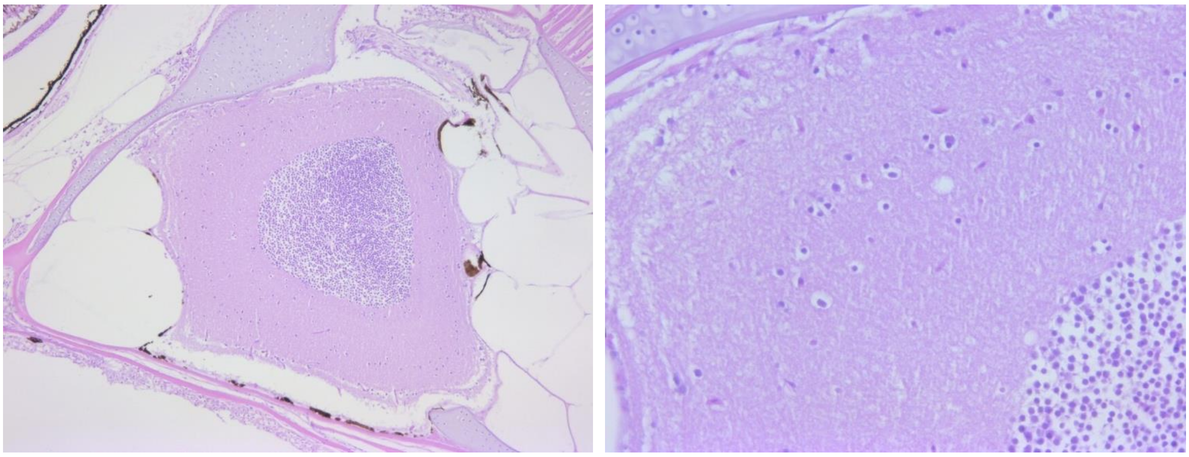

Figure S.2 Histological section of the brain of a guppy male, four days after the end of a heatwave exposure, showing only mild gliosis and perineuronal edema. H&E staining: A and B 10X ob. magnification; C and D 40X ob. magnification.

S.3 Model descriptions

Table S.3 Description of the statistical models used, including the response variables, predictor variables, random effects, model type and family, and sample sizes (N) of the heatwave (HT) and control (C) groups. An observation-level random effect (OLRE) was included to the base models when overdispersion was detected. The random effect BLOCK was removed from models when not significant (indicated in grey), which, in three cases (detour test models) led to the use of non-mixed models (GLM and LM). Interactions were never removed from the models as they were inherent to the experimental design.

	Model			Type	Family	Link	N HT	N C
**Maze test**							HT	C
Search time	log(SearchTime)	~	TREATMENT * TRIAL NUMBER + (1| MALE ID) + (1| BLOCK)	LMER	Gaussian		36	38
Solving probability	SolvedTrial	~	TREATMENT * TRIAL NUMBER + (1| MALE ID) + (1| BLOCK)	GLMER	Binomial	log	36	38
Number of errors	NrErrors	~	TREATMENT * TRIAL NUMBER + (1| MALE ID) + (1| BLOCK) *+ (1|OLRE)*	GLMER	Poisson	logit	36	38
**Mate choice** [1]								
Sexual interest	cbind (TimeC, TimeNC)	~	TREATMENT + FEMALE DIFF + (1| BLOCK) *+ (1|OLRE)*	GLMER	Binomial	log	48	51
Preference strength	cbind (TimePref, TimeNotPref)	~	TREATMENT + FEMALE DIFF + (1| BLOCK) *+ (1|OLRE)*	GLMER	Binomial	log	48	51
Pref. consistency	NrSwitches	~	TREATMENT + FEMALE DIFF + (1| BLOCK) *+ (1|OLRE)*	GLMER	Poisson	logit	48	51
**Detour**								
Solving probability	SolvedTrial	~	TREATMENT + (1| BLOCK)	GLMER	Binomial	log	46	47
Correct probability	CorrectTrial	~	TREATMENT + (1| BLOCK)	GLMER	Binomial	log	46	47
Time barrier	TimeBarrier	~	TREATMENT + (1| BLOCK)	LMER	Gaussian		46	47
**Open field** [2]								
Swimming speed	SwimSpeed	~	TREATMENT * STIMULUS + (1| MALE ID) + (1| BLOCK)	LMER	Gaussian		46	44
Freeze time	cbind (TimeFreez, TimeMove)	~	TREATMENT * STIMULUS + (1| MALE ID) + (1| BLOCK) *+ (1|OLRE)*	GLMER	Binomial	log	46	44
Space use	cbind (SpaceUse, NoUse)	~	TREATMENT * STIMULUS + (1| MALE ID) + (1| BLOCK)	GLMER	Binomial	log	46	44
**Habituation**								
Swimming speed	log(SwimSpeed)	~	TREATMENT * STIMULUS + (1| MALE ID) + (1| BLOCK)	LMER	Gaussian		35	36
Freeze time	cbind (TimeFreez, TimeMove)	~	TREATMENT * STIMULUS + (1| MALE ID) + (1| BLOCK) *+ (1|OLRE)*	GLMER	Binomial	log	35	36

[1] *TimeC:* *Time spent in the choice area*
TimeNC: *Time spent in the non-choice area*
TimePref: *Time with preferred female*
TimeNotPref: *Time with not preferred female*

[2] *TimeFreez:* *Time spent freezing*
TimeMove: *Time not freezing*
SpaceUse: *Number of areas visited*
NoUse: *Number of areas not visited*

S.4 Model-based estimates

Table S.4 Back-transformed estimated marginal means, standard errors (SE) and 95% confidence intervals extracted from the models using the package emmeans (Length, 2021).

			CONTROL				HEATWAVE			
	**Model**	**Type of estimate**	**Mean**	**SE**	**LowerCI**	**UpperCI**	**Mean**	**SE**	**LowerCI**	**UpperCI**
**Maze test**	*Solving prob.*	slope: % change per trial	5.232	10.130	-13.757	28.351	-19.184	9.560	-34.734	-2.537
	Search time	slope: % change per trial	-3.748	1.960	-7.392	0.034	1.918	2.020	-2.049	6.034
	Nr of errors	slope: % change per trial	-5.380	2.500	-9.905	-0.626	1.877	2.700	-3.372	7.418
**Mate choice**	*Sexual interest*	% choice time	96.374	0.008	94.480	97.611	94.268	0.012	91.451	96.231
	Pref. strength	% time preferred female	88.391	0.026	82.361	92.484	74.838	0.047	64.726	82.920
	Switch rate	N switches per minute	0.559	0.100	0.383	0.818	0.823	0.148	0.560	1.207
**Detour**	*Solving prob.*	probability solved trial	0.851	0.052	0.719	0.928	0.870	0.050	0.739	0.940
	Correct prob.	probability correct trial	0.106	0.045	0.045	0.231	0.196	0.059	0.105	0.335
	Time barrier	seconds in front of barrier	590	70	451	729	475	71	335	616
**Open field**	*Swimming speed*	before cue speed (mm/s)	44.9	2.2	40.6	49.3	43.5	2.1	39.3	47.7
		after cue speed (mm/s)	24.0	2.3	19.4	28.6	23.6	2.2	19.3	28.0
	Freeze rate	before cue % freeze time	1.495	0.688	0.606	3.651	1.213	0.549	0.497	2.928
		after cue % freeze time	53.668	11.627	31.648	74.326	44.522	11.111	24.974	65.971
	Space use	before cue % space used	93.214	1.550	89.473	95.689	96.832	0.765	94.926	98.016
		after cue % spece used	75.213	4.495	65.340	82.920	80.690	3.725	72.332	86.989
**Habituation**	*Swimming speed*	before dist. speed (mm/s)	19.106	2.767	14.296	25.534	19.298	2.768	14.440	25.534
		short dist. speed (mm/s)	4.482	0.627	3.387	5.930	4.482	0.638	3.387	5.930
		long dist. speed (mm/s)	4.437	0.658	3.320	5.930	3.975	0.591	2.945	5.312
	Freeze time	before dist.% freeze time	1.088	0.616	0.359	3.261	2.820	1.518	0.972	7.944
		short dist.% freeze time	94.321	2.791	85.680	97.896	93.643	3.161	83.876	97.634
		long dist.% freeze time	88.288	5.726	71.809	95.730	91.293	4.431	77.868	96.923

S.5 The interplay between treatment and female size in male choice

To test whether heatwave exposure alters the effect of female size differences on male choosiness, the interaction between treatment and female size difference was included in the mate choice models. The interaction significantly affected male sexual interest (χ^2 = 4.775, P = 0.029, Figure S5.A), had a marginally non-significant effect on preference consistency (χ^2 = 3.586, P = 0.058, Figure S5.C), and had no effect on preference strength (χ^2 = 0.618, P = 0.432, Figure S5.B).

Post-hoc analysis of the interactions showed that a greater size difference between the two females led to a higher relative time spent in the choice zone, i.e. a higher sexual interest, in control males (post-hoc: z = 3.019, P = 0.003), but not in heatwave males (post-hoc: z = 0.391, P = 0.696). Similarly, a larger female size difference resulted in higher preference consistency, i.e. a lower switch rate, in control males (post-hoc: z = -3.042, P = 0.003), but not in heatwave-exposed males (post-hoc: z = -0.984, P = 0.328).

These results suggest that heatwave-exposed males were less responsive to the magnitude of the female size difference than control males.

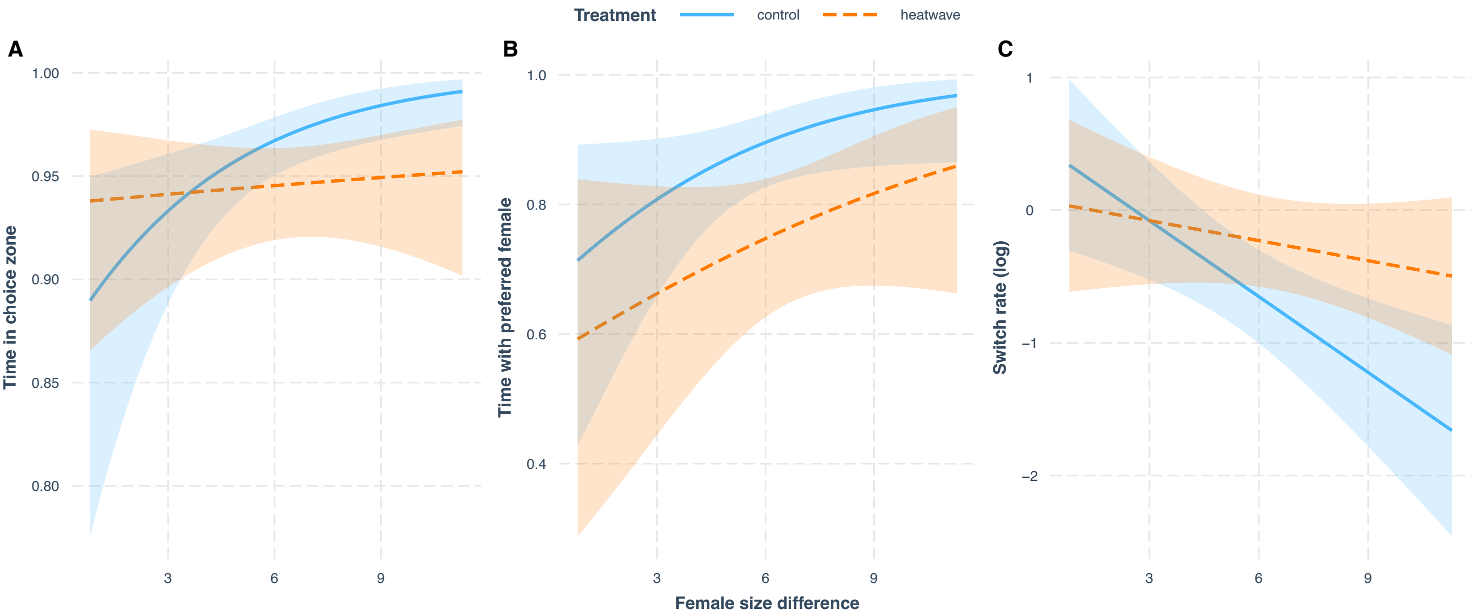

Figure S5. Plot showing the interaction between treatment (control or heatwave) and female size difference on (A) male sexual interest, (B) preference strength, and (C) preference consistency.
